# Activation of the Absent in Melanoma 2 Inflammasome in Peripheral Blood Mononuclear Cells From Idiopathic Pulmonary Fibrosis Patients Leads to the Release of Pro-Fibrotic Mediators

**DOI:** 10.3389/fimmu.2018.00670

**Published:** 2018-04-05

**Authors:** Michela Terlizzi, Antonio Molino, Chiara Colarusso, Chantal Donovan, Pasquale Imitazione, Pasquale Somma, Rita P. Aquino, Philip M. Hansbro, Aldo Pinto, Rosalinda Sorrentino

**Affiliations:** ^1^Department of Pharmacy, University of Salerno, Fisciano, Salerno, Italy; ^2^ImmunePharma s.r.l., University of Salerno, Fisciano, Salerno, Italy; ^3^Respiratory Division, Department of Respiratory Medicine, University of Naples Federico II, Naples, Italy; ^4^PhD Program in Drug Discovery and Development, Department of Pharmacy, University of Salerno, Fisciano, Italy; ^5^Priority Research Centre for Healthy Lungs, Hunter Medical Research Institute, University of Newcastle, Newcastle, NSW, Australia; ^6^Department of Anatomy and Pathology, Ospedale dei Colli “Monaldi-CTO”, Naples, Italy

**Keywords:** idiopathic pulmonary fibrosis, absent in melanoma 2 inflammasome, IL-1α, IL-18, caspase-4

## Abstract

Idiopathic pulmonary fibrosis (IPF) is a chronic fibro-proliferative disease characterized by poor prognosis, with a mean survival of ~2–3 years after definite diagnosis. The cause of IPF is still unknown but it is a heterogeneous condition in which the aberrant deposition of extracellular matrix leads to extensive lung remodeling. This remodeling is a consequence of inflammatory responses, but the mechanisms involved are poorly understood. In this study, we first analyzed a bleomycin-induced mouse model, which showed that higher expression of IL-1β, but not IL-18, was correlated to pulmonary cell infiltration and fibrosis. Then, we found that peripheral blood mononuclear cells (PBMCs) from IPF patients released IL-1α and IL-18 in a NLRP3- and calpain-independent manner after LPS ± ATP stimulation. Instead, the activation of the absent in melanoma 2 (AIM2) inflammasome induced the release of IL-1α in a caspase-1-/caspase-8-independent manner; whereas IL-18 release was caspase-1 dependent. These effects correlated with the release of the pro-fibrotic TGF-β, which was induced by AIM2 activation in a caspase-1- and TLR4-independent manner, but dependent on IL-1α. In this context, the activation of AIM2 induced the release of caspase-4 from IPF-derived PBMCs, which correlated with the mRNA levels of this caspase that was higher in IPF than in healthy PBMCs. In conclusion, our findings identify a novel molecular mechanism whereby the activation of AIM2 could lead to the activation of the non-canonical inflammasome (caspase-4 dependent) that induces the release of IL-1α responsible for the release of TGF-β from PBMCs of IPF patients.

## Introduction

Idiopathic pulmonary fibrosis (IPF), a severe form of interstitial disease, is a chronic and irreversible respiratory disease characterized by inflammation and fibrosis (1, 2). Despite the etiology of IPF is still not known, genetic factors, infections, inhalation of fibers and particles (cigarette smoke and asbestos), and gastroesophageal reflux were suggested as possible risk factors for the development of pulmonary fibrosis (3). Nevertheless, dysfunction in the process of tissue repairing of epithelial cells (4), fibroblast activation, and differentiation into myofibroblasts (5) with the ensuing extensive extracellular matrix deposition, are key processes involved in the fibrotic process (4, 5). In this context, although postulated, the role of chronic inflammation still remains elusive. Recent data suggest that the pathophysiology of IPF is more related to fibroblast dysfunction than to dysregulated inflammation (6). In support, no pharmacological modulation of the immune system seems to be as effective (7). In contrast, human data support the role of inflammation in that pro-inflammatory cytokines and immune infiltrates characterize IPF-derived lungs (8). Moreover, repeated danger stimuli (i.e., silica, asbestos) trigger chronic inflammatory patterns that lead to progressive decline of pulmonary function and architecture (2). In this context, because danger stimuli are well-known activator of Nod-like receptors (NLR) which are involved in the inflammasome activation, the goal of this study was to evaluate the involvement of this latter complex. The inflammasome is a multiprotein complex that comprises the assembly of NLRs or HIN200 family receptors, such as absent in melanoma 2 (AIM2), able to bind the adaptor apoptosis-associated speck-like protein containing a carboxyterminal domain that induces the auto-cleavage of caspase-1 and the activation of IL-1-like cytokines (9). Alternative, non-canonical inflammasomes have also been described, which engage caspase-8 or caspase-11 (also known as caspase-4 in humans) (9, 10), which in turn induce inflammasome-dependent caspase-1 activation and inflammasome independent, pyroptosis-like cell death, *via* the release of such “alarmins” as IL-1α (9).

To note, IL-1β and IL-18, related to the inflammasome activation, have been associated with the development of IPF (11). During acute exacerbation of IPF, alveolar macrophages (AMs) and type II pneumocytes produce high levels of IL-1β in a mouse model of bleomycin-induced fibrosis (12). Moreover, the levels of mRNA for IL-1β and IL-18 were augmented in AMs isolated by bronchoalveolar lavage (BAL), and lung, respectively, from the same patients (13). In addition, IL-1β is also able to induce the production of TGF-β, thus it is plausible that the inflammasome is involved in fibroblast activation and differentiation into myofibroblasts (14).

In this study, we found that IPF-derived peripheral blood mononuclear cells (PBMCs) were able to release IL-1α and IL-18 after the stimulation with Poly dA:dT, an AIM2 ligand. In particular, we found that the activation of AIM2 led to IL-18 release in a canonical manner, in that Ac-Y-VAD-cmk, caspase-1 inhibitor, inhibited the release of IL-18 after Poly dA:dT administration. In contrast, the release of IL-1α was not caspase-1 or caspase-8 dependent, implying the involvement of caspase-4 as non-canonical pathway. Indeed, the activation of AIM2 led to the extracellular release of caspase-4 that was not correlated to cell death, rather to IL-1α release, responsible for TGF-β secretion in IPF-derived PBMCs.

## Materials and Methods

### Animal Model

All animal procedures were approved by the Animal Care and Ethics Committee at the University of Newcastle, Australia. Experimental pulmonary fibrosis was induced by intranasal administration of one dose of bleomycin sulfate (MP Biomedical) to 6–8 weeks Balb/C mice, 0.05 U/mouse, as previously described (15). Control groups received an equal volume of sterile PBS. Tissue collection was performed 28 days after bleomycin treatment.

### Bronchoalveolar Lavage Fluid (BALF)

Mouse multi-lobed lungs were tied off, and BALF was collected from the single left lung lobe by washing with PBS (2 × 500 µl). Cells were pelleted (150 *g*, 5 min) and resuspended in red cell lysis buffer. Remaining cells were cytocentrifuged (300 *g*, 10 min) onto microscope slides. BALF slides were stained with May–Grunwald–Giemsa, and differential counts were enumerated according to morphological criteria using light microscopy as previously described (15).

### Human Samples

We used blood from healthy volunteers and IPF patients recruited at the “Monaldi-Azienda Ospedaliera (AORN)-Ospedale dei Colli” Hospital in Naples, Italy, after their approval according to the Review Board of the hospital and the patients’ informed consent. In addition, all experimental protocols were, as stated above, approved and performed in accordance with the guidelines and regulations provided by the Review Board (protocol n. 422/2017). All the subjects were 50 ± 10 years of age and had no history of allergic diseases or chronic respiratory conditions. Blood was collected and used within 24 h.

### Isolation of Human PBMCs

Peripheral blood mononuclear cells were isolated according to Ficoll’s protocol as already reported (16). Briefly, blood (5 ml) was mixed with cell medium (5 ml) supplemented with sole antibiotics and Ficoll medium (Life Sciences, Italy). PBMCs layer was collected and platelets were separated by centrifugation at 149 *g* for 10 min. PBMCs were then collected in cell medium, plated, and treated for 1, 3, 5, or 24 h accordingly. PBMCs were treated with the following substances: LPS 0.1 µg/ml, ATP 0.5 mM, Ac-Y-VAD-cmk (y-VAD) 1 µg/ml, glybenclamide (Gly) 1 µM, MDL28170 (MDL) 10 µM, Poly dA:dT (dA:dT) 1 µg/ml, z-IETD-fmk (IE) 0.5 µg/ml, nintedanib (10 nM), and pirfenidone (0.1 µg/ml).

### Cytokine Measurements

IL-1α, IL-18, and TGF-β were measured in cell-free supernatants using commercially available enzyme-linked immunosorbent assay kits (ELISAs) (eBioscience, CA, USA; R&D Systems, MN, USA). The released form of caspase-4 was analyzed using a kit patented by ImmunePharma s.r.l. (RM2014A000080 and PCT/IB2015/051262) (Department of Pharmacy, University of Salerno, Italy).

### LDH Levels

The levels of lactate dehydrogenase were measured by using a commercially available kit (Sigma, Italy) following the manufacturer’s instructions. Data were expressed as OD values.

### RT-PCR

Total RNA was isolated from PBMCs by using the RNA extraction kit according to the manufacturer’s instructions (Qiagen, Milan, Italy). Reverse transcription was performed by using first-strand cDNA synthesis kit (Qiagen, Milan, Italy) followed by PCR. Thermal cycling conditions were as follow: 5 min at 95°C, followed by 45 cycles of 30 s at 95°C, 60 s at 58°C, and 30 s at 68°C. Primer pairs were as follow:

Caspase-4→Forward 5′-TCCCTGGGCAAAGATTTCCT-3′
Reverse 5′-GTCCAGCCTCCATATTCGGA-3′
β-actin→Forward 5′-ACTCTTCCAGCCTTCCTTCC-3′
Reverse 5′-CGTACAGGTCTTTGCGGATG-3′

In other set of experiments, we used whole mouse lungs, which were excised and homogenized using a tissue stick homogenizer (BioSpec). Total RNA was extracted using TRIzol (Invitrogen) according to the manufacturer’s instructions. RNA (1 µg) from whole lungs was reverse transcribed using Bioscript (Bioline) and random hexamer primers (Invitrogen). The mRNA expression of IL-1β and IL-18 was determined using real-time PCR (Biorad) and compared with the reference gene hypoxanthine-guanine phosphoribosyltransferase (HPRT) as previously described (17). Primer pairs were as follow:

IL-1β →Forward 5′-TGGGATCCTCTCCAGCCAAGC-3′
Reverse 5′-AGCCCTTCATCTTTTGGGGTCCG-3′
IL-18→Forward 5′-TCAGACAACTTTGGCCGACT-3′
Reverse 5′-CAGTCTGGTCTGGGGTTCAC-3′
HPRT→Forward 5′-AGGCCAGACTTTGTTGGATTTGAA-3′
Reverse 5′-CAACTTGCGCTCATCTTAGGCTTT-3′

### Flow Cytometry Analysis

NLRP3 and AIM2 expression was performed by flow cytometry (BD FacsCalibur, Milan, Italy) using the following antibodies: NLRP3-FITC, AIM2-FITC, and CD14-PE (eBioscience, CA, USA).

### Statistical Analysis

Data are reported as the median ± interquartile range. Statistical differences were assessed with one-way ANOVA followed by multiple comparison post-test or Student’s *t*-test (where appropriate) followed by Mann–Whitney test. *p*-Values less than 0.05 were considered as significant.

## Results

### Lungs of Bleomycin-Induced Fibrosis Express Higher Levels of IL-1β

**Figure 1:**
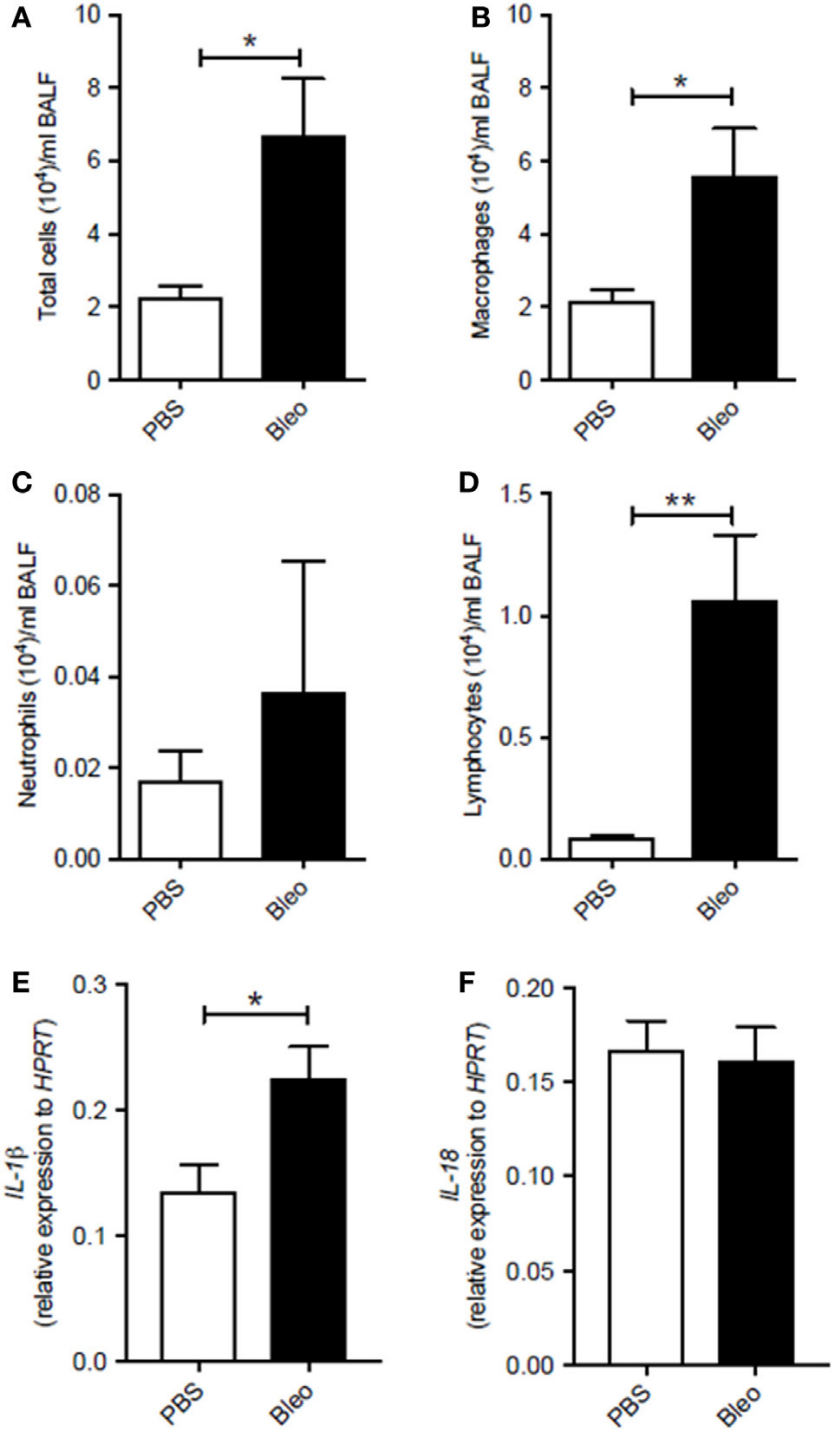
Murine lungs of bleomycin-induced fibrosis express higher levels of IL-1β. Intranasal administration of one dose of bleomycin sulfate (0.05 U/mouse) to Balb/C mice increased **(A)** bronchoalveolar lavage fluid (BALF) total cell count (*p* = 0.019), **(B)** macrophages (*p = 0.019), **(C)** neutrophils (*p* = 0.876), and **(D)** lymphocytes (**p = 0.0095). Analysis of mRNA levels of **(E)** IL-1β (*p = 0.0198) and **(F)** IL-18 (*p* = 0.8159) in the whole lung of bleomycin-treated mice. Data are represented as means ± SEM (*n* = 4–8). Statistically significant differences were determined by Mann–Whitney test for BALF and unpaired Student’s *t*-test for mRNA analysis.

To understand the role of the inflammasome in pulmonary fibrosis, we used an experimental pulmonary fibrosis, induced by intranasal administration of one dose of bleomycin sulfate (0.05 U/mouse) to Balb/C mice, as previously described (15). Control groups received an equal volume of sterile PBS. BALF showed that lungs of fibrotic bleomycin-treated mice had higher cell count (total cells (×10^4^): PBS 2.23 ± 0.31; Bleo 6.71 ± 2.79) than PBS-treated mice (Figure 1A). Differential counts of BALF-derived cells showed that bleomycin-treated mice were characterized by higher presence of macrophages (Figure 1B), neutrophils (Figure 1C), and lymphocytes (Figure 1D) at 28 days after bleomycin treatment. Interestingly, the mRNA expression levels of IL-1β (Figure 1E), but not of IL-18 (Figure 1F), was significantly higher (*p* = 0.0198; PBS 0.14 ± 0.02; Bleo 0.23 ± 0.03) in the whole lung of bleomycin-treated mice than PBS-treated mice, implying the involvement of the inflammasome in this experimental model.

### Human PBMCs From IPF Patients Release IL-1α in an NLRP3 Inflammasome-Independent Manner

**Figure 2:**
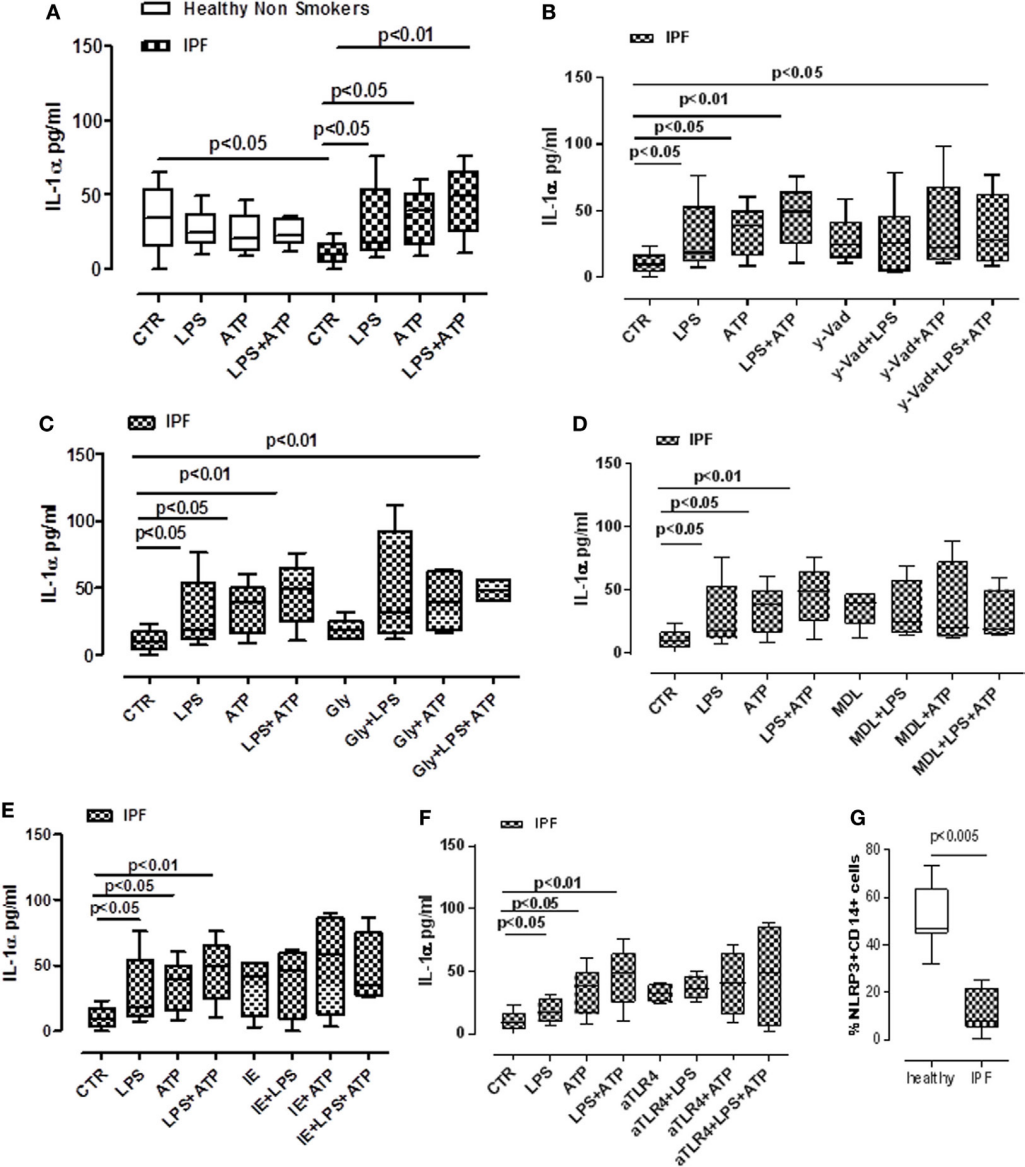
Idiopathic pulmonary fibrosis (IPF)-derived peripheral blood mononuclear cells (PBMCs) release IL-1α in a NLRP3/caspase-1-/caspase-8-/TLR4- and calpain-independent manner. LPS (0.1 μg/ml) ± ATP (0.5 mM) stimulation (5 h) of PBMCs obtained from IPF patients, significantly increased IL-1α release compared with control; no effect was observed in healthy subjects-derived cells after LPS ± ATP addition **(A)**. **(B)** The inhibition of caspase-1 by means of Ac-Y-VAD-cmk (y-VAD, 1 µg/ml) did not induce a reduction of IL-1α release, even after LPS and/or ATP addition. IL-1α levels were not altered after the addition of glybenclamide (Gly, 1 µM) **(C)** or MDL28170 (MDL, 10 µM) **(D)** to LPS ± ATP compared with positive control. Similarly, the inhibition of caspase-8 by means of z-IETD-fmk (IE, 0.5 µg/ml) **(E)** and the blockade of TLR4 **(F)** did not alter the release of IL-1α after dA:dT addition. **(G)** Expression of NLRP3 in CD14^+^ PBMCs. Data are represented as median ± interquartile range (*n* = 13). Statistically significant differences were determined by one-way ANOVA followed by Bonferroni’s multiple comparison post-test or by Student’s *t*-test and Fischer’s exact test as appropriate.

The role of inflammasomes has been partially described in chronic respiratory diseases (10), but its role in IPF is still not clear. To investigate the potential role of these complexes in this pathology, PBMCs from non-IPF healthy volunteers, and IPF age-matched patients were isolated. We observed that the stimulation of cells with LPS (0.1 µg/ml), a TLR4 ligand that induces NLRP3 activation according to the two-signal model (9), induced a significant release of IL-1α from IPF-derived PBMCs (Figure 2A). Conversely, the same effect was not evident for PBMCs derived from healthy individuals, although the basal levels of IL-1α were higher compared with IPF controls (Figure 2A). Interestingly, treatment with LPS + ATP, NLRP3 activators, significantly (*p* < 0.01) increased the levels of IL-1α release from IPF but not from healthy PBMCs (Figure 2A), implying the involvement of NLRP3 in IL-1α release from IPF-derived PBMCs. To understand the involvement of the NLRP3/caspase-1-dependent inflammasome in the release of this cytokine, we tested the effect of Ac-Y-VAD-cmk (y-Vad, 1 µg/ml), a well-known caspase-1 pharmacological inhibitor (18) and Gly (1 µM), an inhibitor of NLRP3 inflammasome (19). Surprisingly, administration of y-Vad did not alter IL-1α release after LPS + ATP treatment (Figure 2B), implying that IL-1α release was not caspase-1 dependent. Similarly, IL-1α levels were not reduced after Gly treatment compared with the positive control (LPS + ATP, Figure 2C), supporting the hypothesis that LPS + ATP-induced IL-1α release was not NLRP3 dependent.

In this context, Gross and colleagues showed that IL-1α was not universally inflammasome dependent. Rather, they showed Rather, they showed that IL-1a secretion was dependent on calpain like proteases, activated by the influx of calcium through the opening of cation channels (20). Therefore, we inhibited the calpain system using MDL28170 (MDL, 10 µM). However, this did not statistically alter IL-1α release (Figure 2D).

To rule out cell death after dA:dT administration, we measured the levels of extracellular LDH. Treatment of IPF-derived PBMCs with dA:dT, in the presence or not of Gly, y-Vad, and MDL, did not alter the levels of LDH among the groups compared with the control (basal) levels (Figure S1 in Supplementary Material).

Moreover, the administration of a caspase-8 inhibitor, z-IETD-fmk (IE, 0.5 µg/ml) did not alter IL-1α levels when IPF-derived PBMCs were treated with LPS ± ATP (Figure 2E).

Because caspase-1 and caspase-8 were not involved in IL-1α release after LPS treatment, and because it was reported that caspase-4 is the intracellular receptor for LPS, we investigated the non-canonical inflammasome pathway (9, 21). To date, no specific inhibitor is actually available to inhibit caspase-4. Therefore, we performed experiments in an indirect manner by inhibiting TLR4, which should allow LPS to enter the cells and induce caspase-4 activation (9, 21). The blockade of TLR4 did not alter IL-1α release from IPF-derived PBMCs after LPS ± ATP addition (Figure 2F), implying the involvement of caspase-4. To confirm our hypothesis, we found that NLRP3 expression in CD14^+^ PBMCs was significantly lower in IPF than healthy subjects (Figure 2G).

Taken together, these data suggest that IPF-derived PBMCs are able to release IL-1α after LPS and/or LPS + ATP stimulation in an NLRP3-/caspase-1-/caspase-8-, TLR4-, and calpain-independent manner, but most likely *via* the involvement of caspase-4.

### Activation of AIM2 Leads to the Release of IL-1α From IPF PBMCs in a Caspase-1- and Caspase-8-Independent Manner

**Figure 3:**
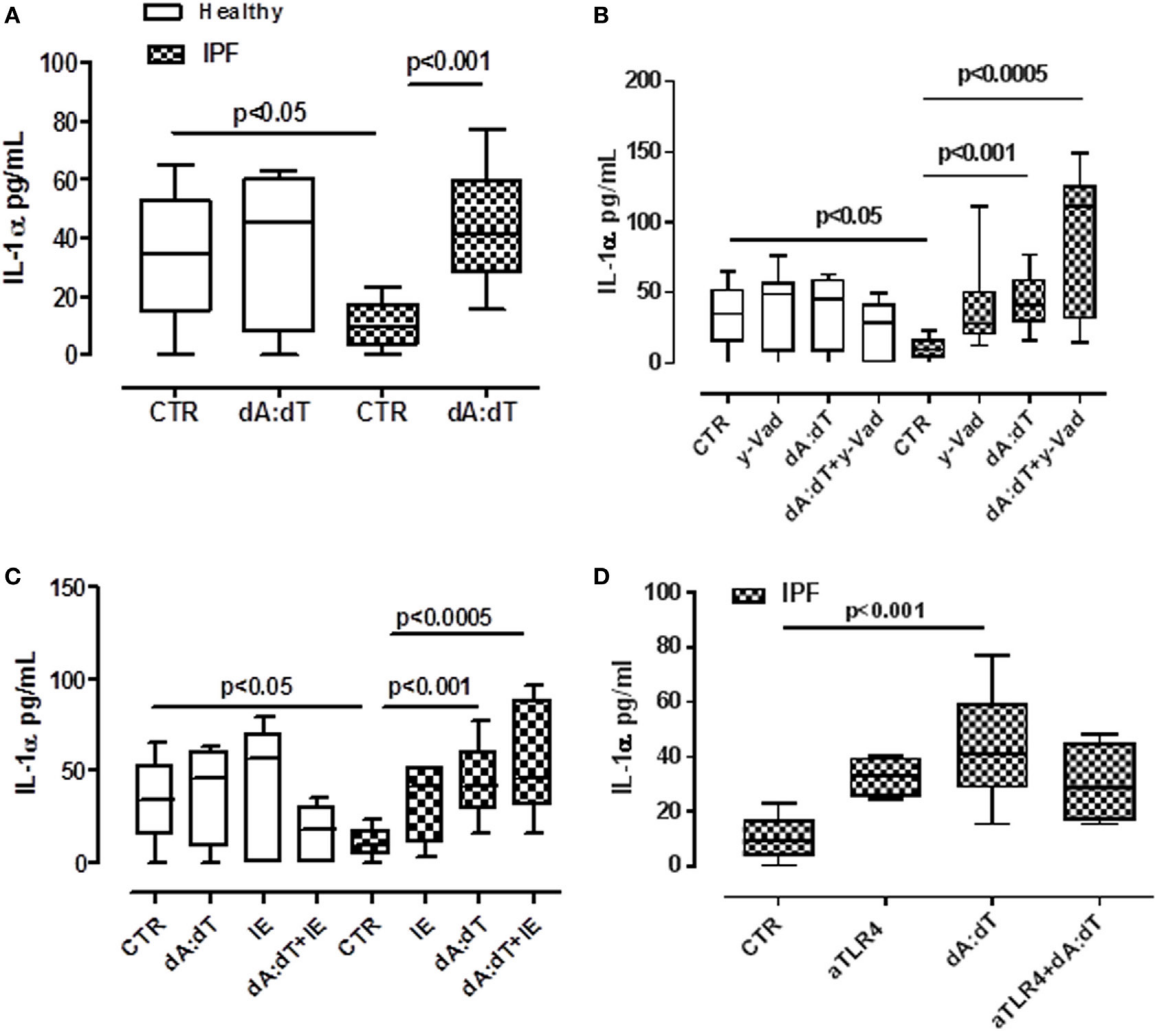
IL-1α release from idiopathic pulmonary fibrosis (IPF)-derived peripheral blood mononuclear cells (PBMCs) is induced after absent in melanoma 2 (AIM2) inflammasome activation. **(A)** Poly dA:dT (dA:dT, 1 µg/ml) stimulation (5 h) of PBMCs obtained from IPF patients, significantly increased IL-1α release compared with control; no effect was observed in healthy subjects-derived cells after dA:dT addition. The inhibition of caspase-1 by means of Ac-Y-VAD-cmk (y-VAD, 1 µg/ml) **(B)** or caspase-8 by means of z-IETD-fmk (IE, 0.5 µg/ml) **(C)** or TLR4 blockade **(D)** did not affect IL-1α release, even after dA:dT treatment. Data are represented as median ± interquartile range (*n* = 13). Statistically significant differences were determined by one-way ANOVA followed by Bonferroni’s multiple comparison post-test.

In our previous study, we demonstrated that the release of IL-1α from lung tumor-associated plasmacytoid dendritic cells was AIM2 inflammasome dependent and promoted tumor cell proliferation in the lung (19). Therefore, we analyzed the possible involvement of AIM2 inflammasome in IL-1α release from IPF-derived PBMCs. The stimulation of PBMCs with Poly dA:dT (dA:dT, 1 µg/ml), an AIM2 ligand, significantly increased IL-1α levels (Figure 3A). Similar to LPS ± ATP, the same effect was not observed from the healthy PBMCs from which IL-1α basal levels were higher than IPF-derived PBMCs (Figure 3A). To understand the molecular mechanism associated with AIM2-dependent IL-1α release, we treated the cells with caspase-1 inhibitor, y-VAD, or caspase-8 inhibitor, IE, in the presence or not of dA:dT. The pharmacological inhibition of caspase-1 (Figure 3B) or caspase-8 (Figure 3C) did not reduce IL-1α levels, rather, its release was further increased compared with treatment with dA:dT alone.

Moreover, because it was described that the intracellular LPS receptor is represented by caspase-4 (9, 21), in order to evaluate whether this enzyme was correlated to IL-1α release, we neutralized TLR4 by means of an antibody and measured the release of IL-1α under dA:dT stimulation. The neutralization of TLR4 did not alter the release of IL-1α after the addition of dA:dT (Figure 3D). We observed the same effect after LPS + ATP stimulation (data not shown).

The isotype control IgG did not alter the levels of basal IL-1α (15.7 ± 2.56 pg/ml, data not shown). These results indicate that AIM2 inflammasome activation in IPF-derived PBMCs leads to IL-1α release in a caspase-1-/caspase-8- and TLR4-independent manner, implying the involvement of another mechanism(s), as observed for LPS ± ATP (Figure 2), most likely due to the non-canonical inflammasome (caspase-4 dependent).

### Activation of AIM2 Leads to the Release of IL-18 From IPF PBMCs in a Caspase-1-Dependent Manner

**Figure 4:**
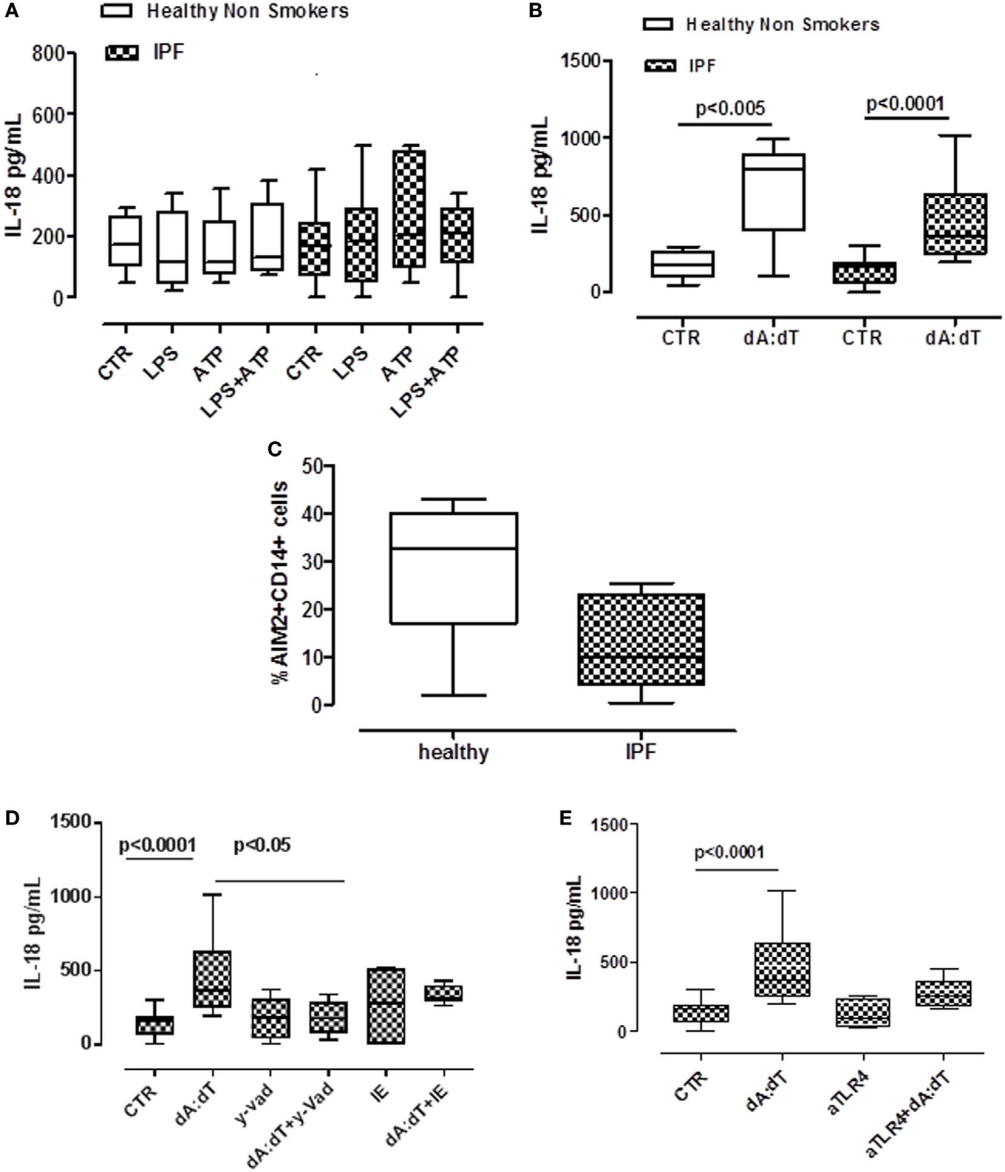
IL-18 release from idiopathic pulmonary fibrosis (IPF)-derived peripheral blood mononuclear cells (PBMCs) is absent in melanoma 2 (AIM2)/caspase-1 dependent. **(A)** LPS ± ATP treatment (5 h) of PBMCs did not induce a significant increase of IL-18 levels both in IPF patients and healthy subjects. **(B)** Poly dA:dT (dA:dT, 1 µg/ml) stimulation (5 h) of PBMCs isolated from IPF patients and healthy subjects significantly increased IL-18 release compared with respective control. **(C)** Expression of AIM2 in CD14^+^ cells. **(D)** Caspase-1 inhibition reduced IL-18 release after dA:dT addition, unlike caspase-8 inhibition. Similarly, TLR4 blockade did not alter IL-18 release after dA:dT administration **(E)**. Data are represented as median ± interquartile range (*n* = 13). Statistically significant differences were determined by one-way ANOVA followed by Bonferroni’s multiple comparison post-test.

Despite a role for IL-18 being reported in many biological processes, its contribution to fibrosis is unclear, with both pro- and anti-fibrotic effects having been demonstrated (11, 22). The addition of LPS or ATP or LPS + ATP did not increase the release of IL-18 from PBMCs from either healthy or IPF patients (Figure 4A). In sharp contrast, IL-18 levels were significantly increased after the stimulation of AIM2 *via* dA:dT administration to both healthy- and IPF-derived PBMCs (Figure 4B). To understand whether AIM2 was expressed, we performed flow cytometry analysis on CD14^+^ PBMCs. We did not observe statistical differences for AIM2 expression between healthy and IPF CD14^+^ PBMCs (Figure 4C).

To define the roles of caspase-1-dependent canonical inflammasome versus caspase-8-dependent non-canonical inflammasome, specific pharmacological inhibitors were used. Contrary to what observed for IL-1α (Figures 3B,C), IL-18 release was abrogated after the addition of y-VAD to dA:dT (Figure 4D); in contrast, the pharmacological inhibition of caspase-8 with IE, did not alter IL-18 levels after dA:dT treatment (Figure 4D) implying that caspase-8 was not involved. Moreover, the inhibition of TLR4 did not alter IL-18 release after dA:dT administration (Figure 4E).

Taken together, these data indicate that IL-18 release from IPF-derived PBMCs was AIM2/caspase-1 dependent following the activation of the canonical inflammasome compared with IL-1α.

### Activation of AIM2 Inflammasome Induces TGF-β Release From IPF-Derived PBMCs in a Non-Canonical Manner

**Figure 5:**
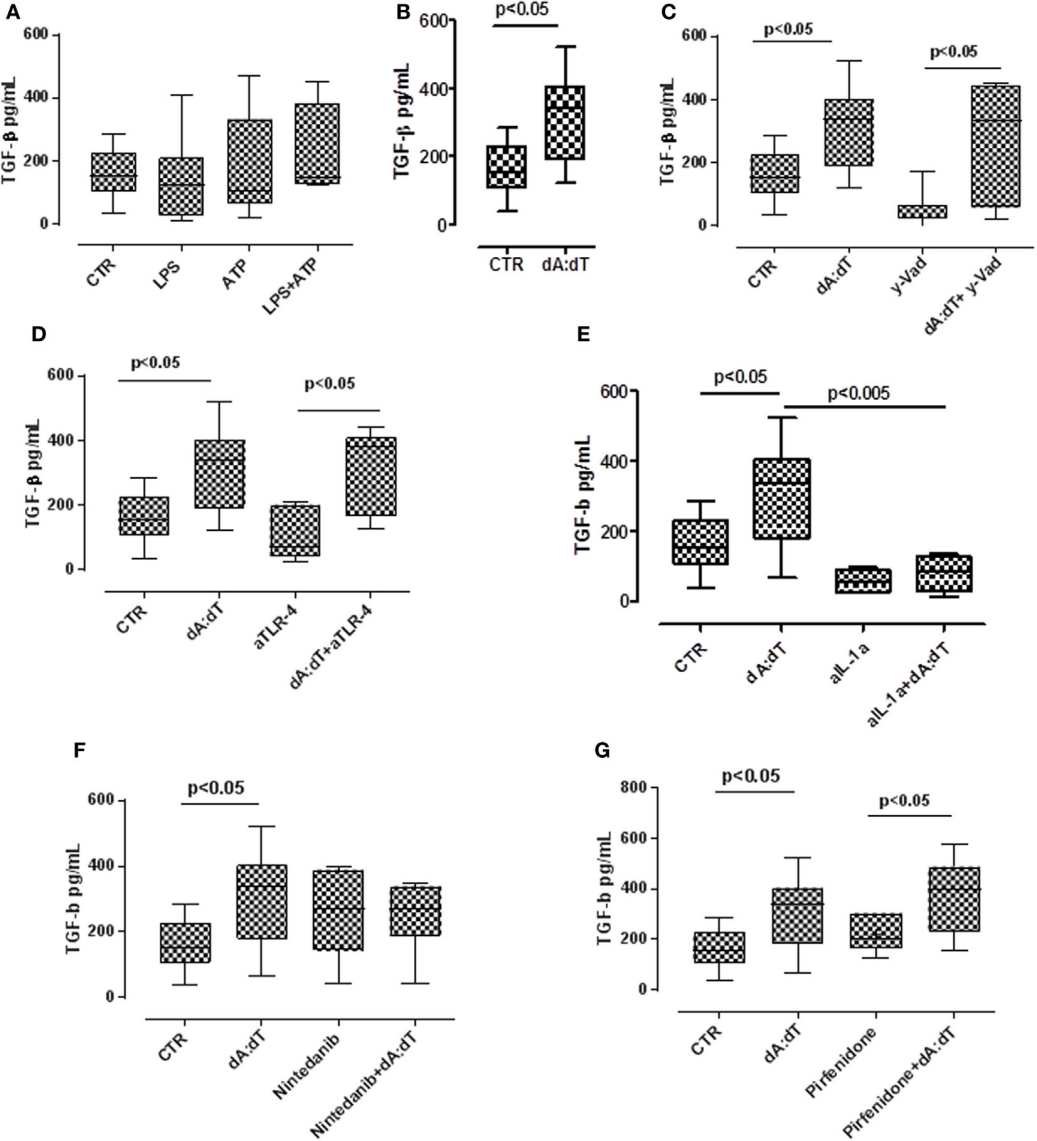
The activation of absent in melanoma 2 inflammasome induces the release of the pro-fibrotic TGF-β. **(A)** Treatment with LPS ± ATP for 24 h induced the release of TGF-β from idiopathic pulmonary fibrosis (IPF) peripheral blood mononuclear cells (PBMCs), although not was a statistical significant manner. **(B)** Poly dA:dT (dA:dT) stimulation significantly increased the release of TGF-β from IPF-derived PBMCs. The inhibition of caspase-1 with Ac-y-Vad (y-Vad) did not reduce TGF-β release after **(C)** dA:dT treatment. The neutralization of TLR4 did not reduce TGF-β release after dA:dT treatment **(D)**. Instead, the neutralization of IL-1α significantly reduced TGF-β release **(E)**. In sharp contrast, the addition of nintedanib (10 nM) **(F)** or pirfenidone **(G)** did not alter dA:dT-induced TGF-β release. Data are represented as median ± interquartile range (*n* = 13). Statistically significant differences were determined by one-way ANOVA followed by Bonferroni’s multiple comparison post-test or Student’s *t* -test and Fischer’s exact test as appropriate.

TGF-β has long been proposed as a key molecule in the pathogenesis of lung fibrosis (23, 24), thus we examined its role in our experimental conditions. We found that the stimulation with LPS + ATP tended (not statistically significant) to increase TGF-β levels (Figure 5A) from IPF-derived PBMCs. In contrast, the stimulation of AIM2 with dA:dT significantly increased the release of TGF-β from IPF-derived PBMCs after 24 h of treatment (Figure 5B).

To examine the molecular mechanisms, we treated the cells with a caspase-1 inhibitor. The inhibition of caspase-1 with y-Vad did not alter TGF-β levels after dA:dT administration (Figure 5C), suggesting that AIM2-induced TGF-β release from IPF patients-derived PBMCs was caspase-1 independent.

It is well known that TLR4 signaling results in augmented TGF-β responses with increased matrix production and progressive connective tissue remodeling (25), and that TLR4 stimulation controls inflammasome activation (9, 26). Moreover, because we already observed that the neutralization of TLR4 did not alter the release of IL-1α after the addition of dA:dT (Figure 3D), we tested the levels of TGF-β in these conditions. We observed that the release of TGF-β after the activation of AIM2 *via* dA:dT was not altered by the neutralization of TLR4 (Figure 5D). The isotype control IgG did not alter the levels of basal TGF-β (107 ± 28 pg/ml, data not shown). In sharp contrast, the neutralization of IL-1α by using a monoclonal antibody (isotype control IgG), significantly reduced the release of TGF-β after dA:dT treatment (Figure 5E), implying that IL-1α is involved in TGF-β release.

To further explore the molecular mechanism that leads to TGF-β release from IPF-derived PBMCs, we treated the cells with the two drugs that are actually used in therapy, nintedanib (10 nM), a tyrosine kinase inhibitor, and pirfenidone (0.1 µg/ml). Surprisingly, neither nintedanib (Figure 5F) nor pirfenidone (Figure 5G) reduced the levels of TGF-β from IPF-derived PBMCs.

Taken altogether, these data imply that the activation of AIM2 leads to IL-1α that promotes TGF-β release from IPF-derived PBMCs.

### Activation of AIM2 Leads to Caspase-4 Release From IPF PBMCs

In our previous data, we showed that the activation of AIM2 with dA:dT led to the release of IL-1α and TGF-β in a caspase-1, caspase-8-, and calpain-independent manner; whereas IL-18 was released in a caspase-1-dependent manner. Moreover, because in our previous data we found that caspase-1, caspase-8, calpain I/II axis, and TLR4 activity were not involved in IL-1α release, we went on by analyzing the involvement of caspase-4.

**Figure 6:**
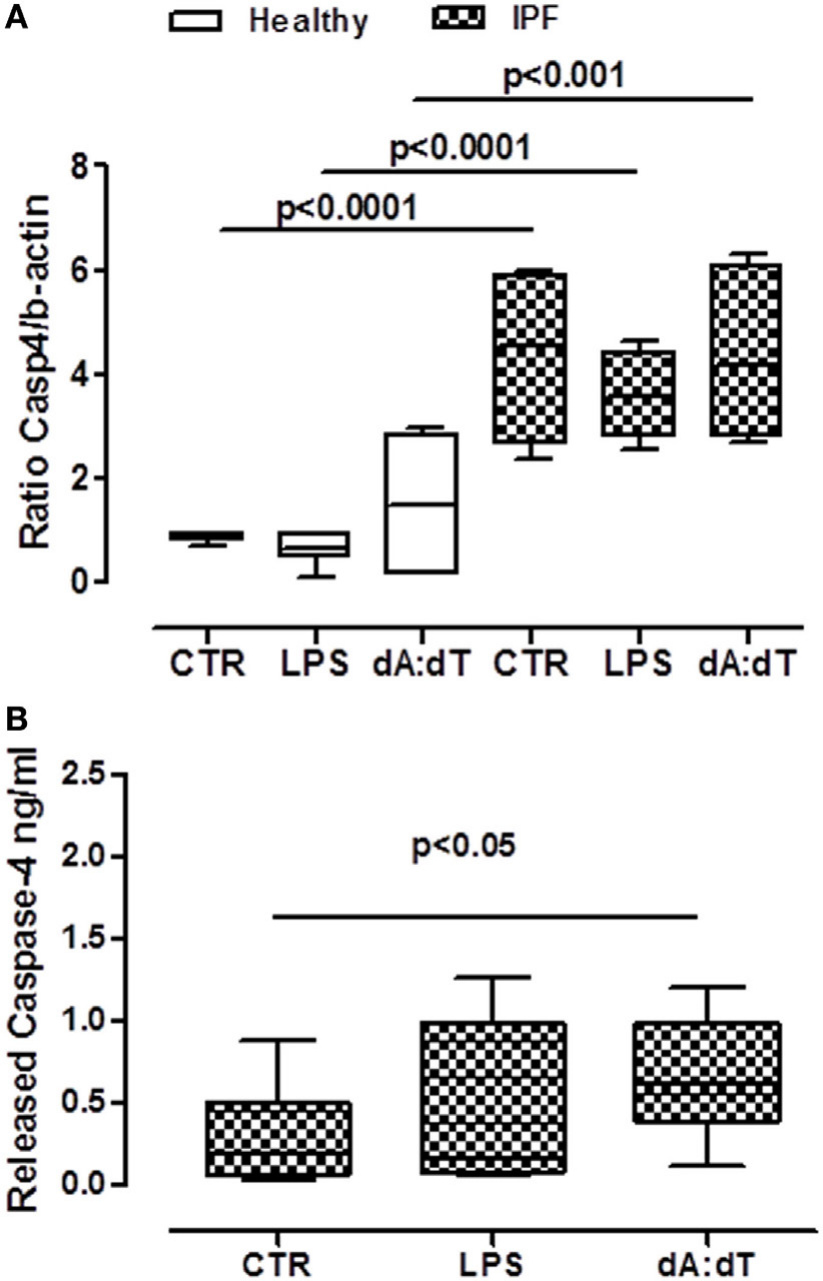
Caspase-4 extracellular release from idiopathic pulmonary fibrosis (IPF) peripheral blood mononuclear cells (PBMCs) after AIM2 inflammasome activation. **(A)** PBMCs derived from IPF patients showed higher basal levels of caspase-4 mRNA compared with healthy subjects. **(B)** Caspase-4 release in the supernatant of IPF-derived PBMCs increased after 5 h of treatment with LPS and dA:dT. Data are represented as median ± interquartile range (*n* = 13). Statistically significant differences were determined by one-way ANOVA followed by Bonferroni’s multiple comparison post-test.

We first analyzed the levels of mRNA for caspase-4 in PBMCs obtained from IPF patients and healthy subjects. Caspase-4 mRNA levels were very low in healthy non-IPF, control cells (0.4 ± 0.31, Figure 6A). In contrast, the levels were noticeably increased in PBMCs from IPF patients (4.357 ± 0.86) (Figure 6A) compared with healthy PBMCs, suggesting an important role for this protease during pulmonary fibrosis. Notably, however, no increases in mRNA levels of caspase-4 were detected after LPS or dA:dT stimulation in IPF PBMCs (Figure 6A). In order to understand the potential involvement of caspase-4 in our experimental conditions, we used a patented ELISA kit that detects the released form of caspase-4. Interestingly, the addition of dA:dT to IPF-derived PBMCs significantly increased the release of caspase-4 (Figure 6B).

**Figure 7:**
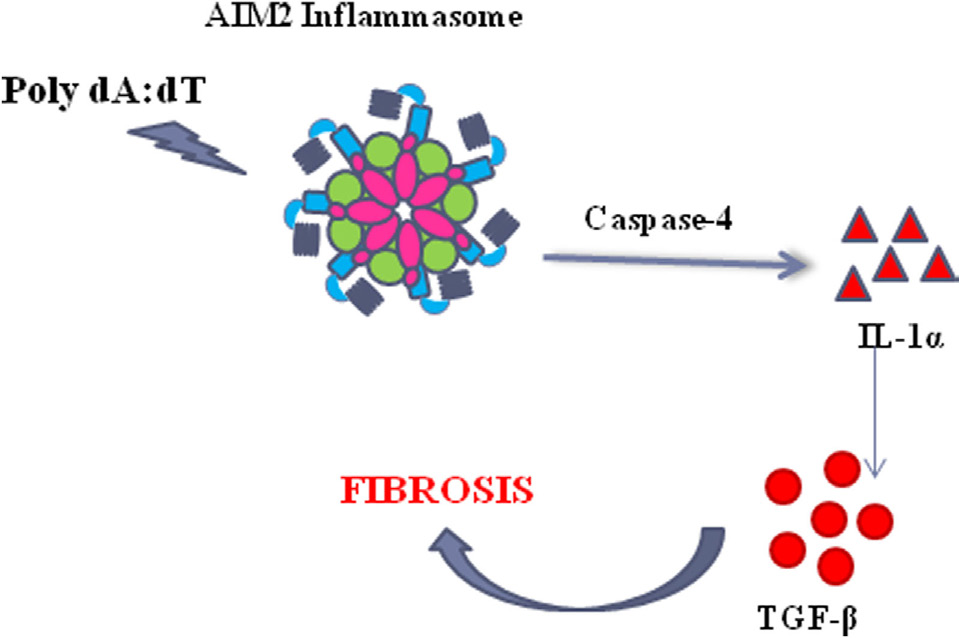
The activation of the absent in melanoma 2 (AIM2) inflammasome induce the release of caspase-4 which leads to IL-1α release, responsible of TGF-β release in idiopathic pulmonary fibrosis-derived peripheral blood mononuclear cells.

Taken together, these data, although not in a direct manner for the absence of a specific inhibitor, suggest that the activation of AIM2 leads to caspase-4 release responsible of IL-1α-dependent pro-fibrotic TGF-β release (Figure 7).

## Discussion

The role of the inflammasome in the development/exacerbation of IPF has been suggested, although it is still elusive. In our study, we found that the activation of AIM2 inflammasome in IPF PBMCs led to IL-1α, but not IL-1β, and TGF-β release in a TLR4-/caspase-1-/caspase-8-/calpain-independent manner, concomitantly to caspase-4 extracellular release. IL-18, instead, as observed in healthy cells, was AIM2/caspase-1 dependent. Notably, treatment of IPF PBMCs with LPS and/or LPS + ATP, well-known activators of NLRP3, led to the release of IL-1α in an NLRP3-/caspase-1/caspase-8-/TLR4- and calpain-independent manner, leading to a slight increase of caspase-4 release.

Several published data report NLRP3 as involved in lung fibrosis in both experimental and human samples. In our study, we found that the levels of NLRP3 were lower in IPF-derived PBMCs, explaining why its activation *via* LPS + ATP was not responsible for both IL-18 and IL-1α release. In addition, in order to further evaluate the role of NLRP3, we used the diazoxide, as an inducer of NLRP3 in that it increases K + influx (19). Similarly, we did not observe an increase in IL-1α and TGF-β (data not shown) from IPF-derived PBMCs, supporting that the lower expression of NLRP3 in IPF-derived PBMCs is not functional. Instead, experimental animal models showed that NLRP3 ko mice were protected from mechanical ventilation- (27) and silica- (28), asbestos-induced pulmonary fibrosis (13). The discrepancy with the latter data and ours may reflect the difference between human and mouse samples. Indeed, in our experimental model of bleomycin-induced pulmonary fibrosis (measured by collagen deposition around the airways) (15), we observed that IL-1β, rather than IL-18, as observed in humans, is upregulated in mRNA levels after bleomycin treatment. In contrast, treatment of human PBMCs with both LPS ± ATP and/or Poly dA:dT did not induce detectable levels of IL-1β (data not shown) in our experimental conditions (5 h of treatment). This result may underlie an impairment in NLRP3 response when LPS ± ATP were added. In support, Lasithiotaki et al. reported that NLRP3 was not involved in IPF patients (29). Similarly, we did not observe any inhibition of IL-1α and IL-18 after the addition of Gly, an inhibitor of NLRP3, implying that these cytokines are not NLRP3-related in IPF PBMCs. In the same manner, the release of these cytokines was not altered after caspase-1/caspase-8 inhibition in IPF-derived PBMCs. Because caspase-8, besides caspase-1, are reported as understream NLRP3 activation, we can conclude that because NLRP3 is not involved in the release of IL-1α and IL-18 in PBMCs of IPF patients, it is likely that LPS triggers the intracellular caspase-4 (9, 21) that is released (although not in a statistically different manner) after LPS addition. Instead, the release of caspase-4 was more evident when AIM2 (upstream) was activated. However, in this context, it has to be noted that the stimulation of healthy PBMCs with LPS ± ATP did not induce the release of IL-1α and IL-18, although a higher expression of NLRP3 in healthy PBMCs. We can speculate that this phenomenon could be related to a protective regulatory pathway that does not occur in IPF PBMCs. Indeed, when comparing healthy and IPF PBMCs, we did not observe substantial differences in the release of IL-1α. After the stimulation with LPS ± ATP or PolydA:dT the levels of IL-1α from IPF-derived PBMCs were comparable with the one from healthy PBMCs. Nevertheless, the basal levels of this cytokine were significantly different, higher in healthy than IPF. However, when IPF PBMCs were stimulated there was an increase from the basal level, compared with the healthy PBMCs, which did not induce any increase in the levels of IL-1α. These data may imply that a regulatory/protective mechanism occurs in healthy PBMCs compared with the pathological condition, during which the same values of the cytokine can contribute to fibrosis.

On the other hand, we observed that the stimulation of another inflammasome receptor AIM2 led to the release of both IL-1α and IL-18, but not of IL-1β (not detectable), from PBMCs. In this case, though, IL-18 release was caspase-1 dependent, compared with IL-1α which instead was caspase-1-, caspase-8 independent. Various studies demonstrated the involvement of IL-18 in the pathogenesis of IPF. IL-18- and IL-18Rα-deficient mice were protected from bleomycin-induced lung fibrosis, suggesting a pro-fibrotic role for IL-18 (30). In addition, Kitasato et al. reported that IL-18 was expressed in IPF pulmonary cells (22). Conversely, other studies suggested a protective role against bleomycin-induced lung fibrosis since IL-18 deficient mice had higher survival rate than wild-type (31).

In our conditions, because the release of IL-18 *via* AIM2 was similar in both healthy and IPF subjects we believe that the most relevant activity of AIM2 during the pathological conditions was related to IL-1α. The involvement of IL-1α in chronic lung diseases has been repeatedly reported (11, 32–34). In particular, this cytokine is involved in the phenotypic switch of lung fibroblasts to their inflammatory state during epithelial damage. Moreover, it has been suggested that extracellular IL-1α is an undesiderable and potentially harmful factor in fibrotic lung diseases (35). In support, IL-1R1−/− and IL-1α−/− mice exhibited reduced BAL neutrophilia and collagen deposition in response to bleomycin treatment (35) confirming the role of circulating anti-IL-1α autoantibodies detected in the blood of IPF patients (36).

In our experimental conditions, IL-1α was neither correlated to the caspase-1- and caspase-8-dependent inflammasome activation, nor to the calpain complex. Rather, it was correlated to AIM2 activation, caspase-4 extracellular release, and TGF-β release.

TGF-β is a well-known pro-fibrotic and immunosuppressive cytokine (23, 24). In IPF, it plays a pivotal role in that it stimulates intrapulmonary fibroblasts to express high levels of collagen genes and mesenchymal cell-related markers, such as α-smooth muscle actin and vimentin (37). Our study is the first, to our knowledge, to show that the activation of the AIM2 inflammasome induces TGF-β release in an IL-1α-dependent manner. The neutralization of IL-1α by means of a monoclonal antibody, significantly reduced the release of TGF-β at 24 h. Differently, two drugs that are actually used in therapy (nintedanib and pirfenidone) did not alter TGF-β release from PBMCs. More importantly, this effect was not dependent on TLR4, rather, it was correlated to caspase-4 release, that was not expression of cell death (no differences in LDH levels after AIM2 activation). In this context, the extracellular release of caspase-4 is associated to cell mortality in literature. Rather, our data are the first, to our knowledge, to show that the activation of AIM2 leads to caspase-4 release in IPF PBMCs, which were not dying cells, but were able to release IL-1α, responsible for TGF β levels. In support, the inhibition of TLR4, did not alter both IL-1α and TGF-β release from IPF PBMCs. Moreover, AIM2 activation *via* Poly dA:dT increased both mRNA and extracellular release of caspase-4. These data, although not a direct evidence, likely correlate caspase-4 to the release of IL-1α.

In conclusion, our study focuses on a novel molecular pro-fibrotic mechanism that underlies the role of AIM2 and caspase-4 in that the activation of AIM2 leads to the release of pro-inflammatory cytokines, such as IL-1α and IL-18, but also to the activation of a non-canonical inflammasome caspase-4-dependent pathway. In particular, we believe that the activation of AIM2 leads to caspase-4 activation/release with the concomitant release of IL-1α responsible of the pro-fibrotic TGF-β levels (Figure 7). Although the strict correlation between these pathways still remains to be elucidated, we propose novel molecular targets to improve survival rate of IPF patients, that unfortunately is still very low, due to the lack of efficacy of several therapeutic tools (i.e., steroids). To our opinion, we highlighted a novel molecular/cellular mechanism that can lead to a pro-fibrotic process in IPF patients, opening new therapeutic perspectives, especially during therapy with nintedanib and pirfenidone that are at the moment the sole therapeutic options for IPF patients.

## Ethics Statement

We used blood from healthy volunteers and Idiopathic Pulmonary Fibrosis (IPF) patients recruited at the “Monaldi-Azienda Ospedaliera (AORN)-Ospedale dei Colli” Hospital in Naples, Italy, after their approval according to the Review Board of the hospital and the patients’ informed consent. In addition, all experimental protocols were, as stated above, approved, and performed in accordance with the guidelines and regulations provided by the Review Board (protocol n. 422/2017).

## Author Contributions

MT, AM, CC, and CD performed the experiments. AM, PI, PS, RA, PH, AP, and RS designed the experimental protocol. PM and RS interpreted the data and wrote the manuscript. All authors read and approved the final manuscript.

## Conflict of Interest Statement

The authors declare that the research was conducted in the absence of any commercial or financial relationships that could be construed as a potential conflict of interest.

## Supplementary Material

Figure S1 LDH levels measured after the addition of dA:dT in the presence or not of glybenclamide (Gly), NLRP3 inhibitor, y-VAD, caspase-1 inhibitor and MDL, calpain I/II inhibitor. Data were expressed as OD values and represented as median ± interquartile range.
