# Supplementary figures and images for: Activation of the Absent in Melanoma 2 Inflammasome in Peripheral Blood Mononuclear Cells From Idiopathic Pulmonary Fibrosis Patients Leads to the Release of Pro-Fibrotic Mediators
Source: Front Immunol. 2018 Apr 5;9:670. doi: 10.3389/fimmu.2018.00670 (PMC5895962; doi:10.3389/fimmu.2018.00670)

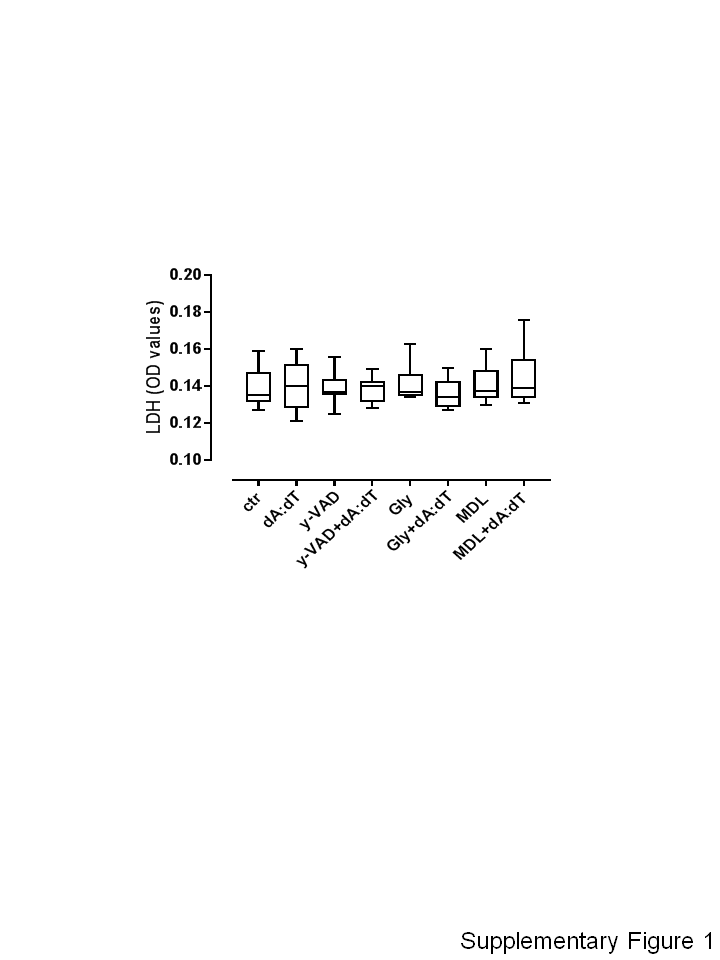

Supplement: Figure S1 — LDH levels measured after the addition of dA:dT in the presence or not of glybenclamide (Gly), NLRP3 inhibitor, y-VAD, caspase-1 inhibitor and MDL, calpain I/II inhibitor. Data were expressed as OD values and represented as median ± interquartile range. [file image_1.TIF]
